# Assessing economic implications for micro, small and medium enterprises in Thailand post Covid-19 lockdown

**DOI:** 10.1371/journal.pone.0294890

**Published:** 2024-02-13

**Authors:** Wanamina Bostan Ali, Joshua Akinlolu Olayinka, Md. Mahmudul Alam, Arno Immelman

**Affiliations:** 1 Business Administration Department, Faculty of Management Sciences, Prince of Songkla University, Hat Yai, Songkhla, Thailand; 2 Logistics Analytics and Supply Chain Management Program, International College, Walailak University, Thasala, Nakhon Si Thamarat Province, Thailand; 3 Economic and Financial Policy Institute, School of Economics, Finance & Banking, Universiti Utara Malaysia, UUM, Sintok, Kedah, Malaysia; 4 Accounting Research Institutes, University Technology Mara, UiTM, Shah Alam, Selangor, Malaysia; Czestochowa University of Technology: Politechnika Czestochowska, POLAND

## Abstract

Micro, Small, and Medium-sized Enterprises (MSMEs) in Thailand were assessed in this study to determine the short-term and long-term economic effects of post-COVID- 19 -, with the goal of developing policy guidelines that focus on the methods and strategies that will further develop and help recover these sectors. MSMEs are the most vulnerable and require assistants to combat the pandemic. This study assesses the perspectives of stakeholders on the development of mechanisms and the strategies applied to support vulnerable groups in Thailand, which mostly consist of women and children. The main data collection was gathered through online questionnaires that were distributed to various stakeholder groups. The tools used for analysis were advanced quantitative analysis tools that aid in achieving this research study’s objectives, and data was examined primarily through the usage of path modeling, structural equation modeling (SEM), and descriptive analysis was among the methods used. The findings reveal that in the short term, MSMEs’ ability to respond to COVID-19 implications has a significant impact on both financial and non-financial performance. Non-financial performance, on the other hand, is more affected by adaptability than financial performance. Demand shock from lockdowns and other COVID-19 cautionary interventions has a negative and significant impact on MSMEs’ adaptability, financial performance, and non-financial performance. The demand shocks increased the vulnerability of MSMEs significantly but it was found that proper management of demand shock has helped stabilized and improve MSMEs’ financial and non-financial performances, as well as helped decrease their vulnerability. When it comes to government policy, the focus is usually on enhancing the flexibility and financial performance of MSMEs. The government’s legislative actions have little impact on MSMEs’ non-financial performance and vulnerability. This could be because the majority of the programs are more focused on providing financial assistance to businesses or their consumers. COVID-19’s supply and demand shock only hindered MSMEs’ ability to respond to the changes and challenges caused by the pandemic, according to vendors. The vulnerability of MSMEs caused by COVID-19 creates grave effects on their financial performance. The findings of this research paper will assist policymakers in identifying the most vulnerable aspects of MSMEs, as well as their expectations- and determine the forms of support that will be required to combat the current and future pandemic situations that may occur in Thailand. In addition, it will aid policymakers in the establishment of procedures and supporting strategies for MSMEs to reduce the unemployment rate and stimulate the Thai economy, among other factors of improvement.

## 1.0 Introduction

In December 2019, several cases of pneumonia of unknown origin were reported in Wuhan, Hubei Province, China, and were later linked to the Huanan Seafood Wholesale Market [1]. The virus, which is now called COVID-19, is caused by a unique coronavirus, labeled SARS-CoV-2, which was discovered through whole genome sequencing, polymerase chain reaction (PCR), and culture of bronchoalveolar lavage fluid obtained from affected patients [1, 2]. This virus, which is the seventh coronavirus that has been proven to infect humans, has 75–80% genomic similarity to the severe acute respiratory syndrome coronavirus (SARS-CoV), 50% to the Middle East respiratory syndrome coronavirus (MERSCoV), and 96% to a bat coronavirus, and uses the same cell receptor, angiotensin-converting enzyme II (ACE2), that is used by SARS-CoV [1, 2]. This is the third coronavirus that has emerged in the past 2 decades, causing multinational outbreaks and carrying substantial morbidity and mortality rates [3, 4].

As of November 29, 2021, over 261 million confirmed COVID-19 cases had been reported to the World Health Organization (WHO). Among them, there were over 5 million deaths. With the expectation that these numbers are likely to increase, there are increasing global concerns about the outbreak, particularly for the intensive care community [5]. This pandemic has served grave consequences for the health and economies of all nations around the world [6]. The pandemic does not only influence the worldwide well-being condition but also the structure of the worldwide economic order. Thus, numerous economies are at the beginning of a downturn [7].

COVID-19 is imperiling the financial situation of people and businesses [8]. In their most recent worldwide financial matters study, Legislative Research Service (2020), revealed the pandemic had reduced worldwide economic development from 0.5% to 1.5% as of March 2020. Ernst and Young (2020) uncovered in their Global Capital Confidence Barometer study that 73% of respondents have experienced a serious effect on the world economy, while the other 27% experienced a minor effect [9]. The broad nearby and cross-border development control, including the collapse of neighborhoods, and public and global business substances, also had an impact on the global economy [10]. Therefore, a large number of laborers are resting under restrictions, and small firms are finding it hard to recover and get back on track [8, 10]. Airlines, the travel industry, travel-related ventures, inns, and cafés are among the most affected and disturbed groups during the Military Controlled Movement (MCO), while staple merchandise makers, groceries, medical services, pharmaceutical, and agro-businesses are less vulnerable and affected [7, 11].

Evidence on the COVID-19 emergency impacts on MSMEs from business studies shows serious disturbances and worries among small enterprises. The magnitude of MSME concerns is affirmed in an ongoing NBER paper that presents the after-effects of an overview of more than 5,800 small businesses in the United States. The study shows that 43% of responding enterprises are now unstable. Overall, businesses have cut down their workforce by 40%. Seventy-five percent of respondents say they have two months or less worth of cash for possible later use. Humphries, Neilson, and Ulyssea (2020) report the tantamount effects of the pandemic on MSMEs [12]. Correspondingly, as per an overview of MSMEs in 132 nations by the International Trade Center, 66% of miniature and small-sized firms report that the crisis of the pandemic unequivocally influenced their business activities, and one-fifth demonstrate the danger of closing down permanently within three months [13],. Several surveys gathered from different nations, demonstrate that somewhere in the range of 25% and 36% of MSMEs could shut down indefinitely from the damages they have experienced in the first four months of the pandemic [8]. In the United States, a particular MSMEs study was set up by the Census Bureau to gauge the effect of COVID-19 on small firms [14]. In late June, the study showed that practically 90% of independent ventures encountered a solid (51%), moderate (38%), or no negative effect from the pandemic; 45% of organizations experienced problems in supply chains; 25% of businesses have under 1-to-2-month worth of cash stored for later use.

The effects of COVID-19 on overall MSMEs’ business involvement are gigantic. Although severe government strategy and reaction to check the infection are important, most MSMEs are exposed to negative impacts in either the short or long period. Significant obstacles are income issues, the closure of activities, laying off workers, conservation, and weakened firms’ ability for future expansion [10, 15]. Changes in business procedures, activities, and business leads, as well as ways to look for new sources and open doors for redevelopment, are perceived as significant endurance challenges for most MSMEs [16, 17]. However, the effects may vary regarding the kinds of business movement, size, and assets possessed [16]. In that capacity, there is a basic need to examine the effect of these factors because currently there is minimal information that is accessible to practitioners, strategy creators, and the scholarly community. Therefore, based on the literature review, there are hardly any impact assessments and survey-based studies of COVID-19’s impact on MSMEs in South East Asia, particularly in Thailand, since the emergence of the COVID-19 pandemic earlier this year. Thus, the need for this study to fill the gap is necessary.

Therefore, this study aims at assessing the influences of COVID-19 on demand shocks and government initiatives on MSMEs’ financial and non-financial business performances in Thailand. This study focuses on MSMEs in Thailand since MSMEs account for a large portion of the total businesses in different sectors. In the manufacturing sector, for instance, SMEs comprise 93.8% of all businesses. Moreover, of the total number of SMEs, small enterprises comprise 76.0%, while medium-sized companies account for 17.8% of all manufacturing businesses. Meanwhile, it is estimated that 90% of all manufacturing businesses were SMEs, employing 38.9% of the total [18]. This emphasizes the importance of MSMEs in terms of poverty mitigation through job creation in Thailand. Thai MSMEs are increasingly seen as creators of new jobs [19] and can compete at regional and international levels. In addition, the Office of Small and Medium Enterprise Promotion of Thailand announced in 2016 that Thailand’s Gross Domestic Product (GDP) expanded by 3.2% for SMEs compared to 2015. It alludes to the great importance of MSMEs for the Thai economy [20]. This study will help Thai MSMEs, the government, and other policymakers understand the effects of COVID-19 from different perspectives and help them take appropriate initiatives to combat current and future problems that will be caused by the COVID-19 pandemic.

## 2.0 Literature review

### 2.1 Business performance impact from the supply side

According to CEPR’s policy portal, the COVID-19 situation has severely disrupted the world economy’s supply side, shutting down entire industries [21]. The coronavirus pandemic affects the economy, particularly MSMEs, in a variety of ways on both the supply and demand sides. On the supply side, companies see a decrease in the supply of labor as workers become ill or need to care for children or other dependents while schools are closed and people’s movements are restricted.

**Fig 1:**
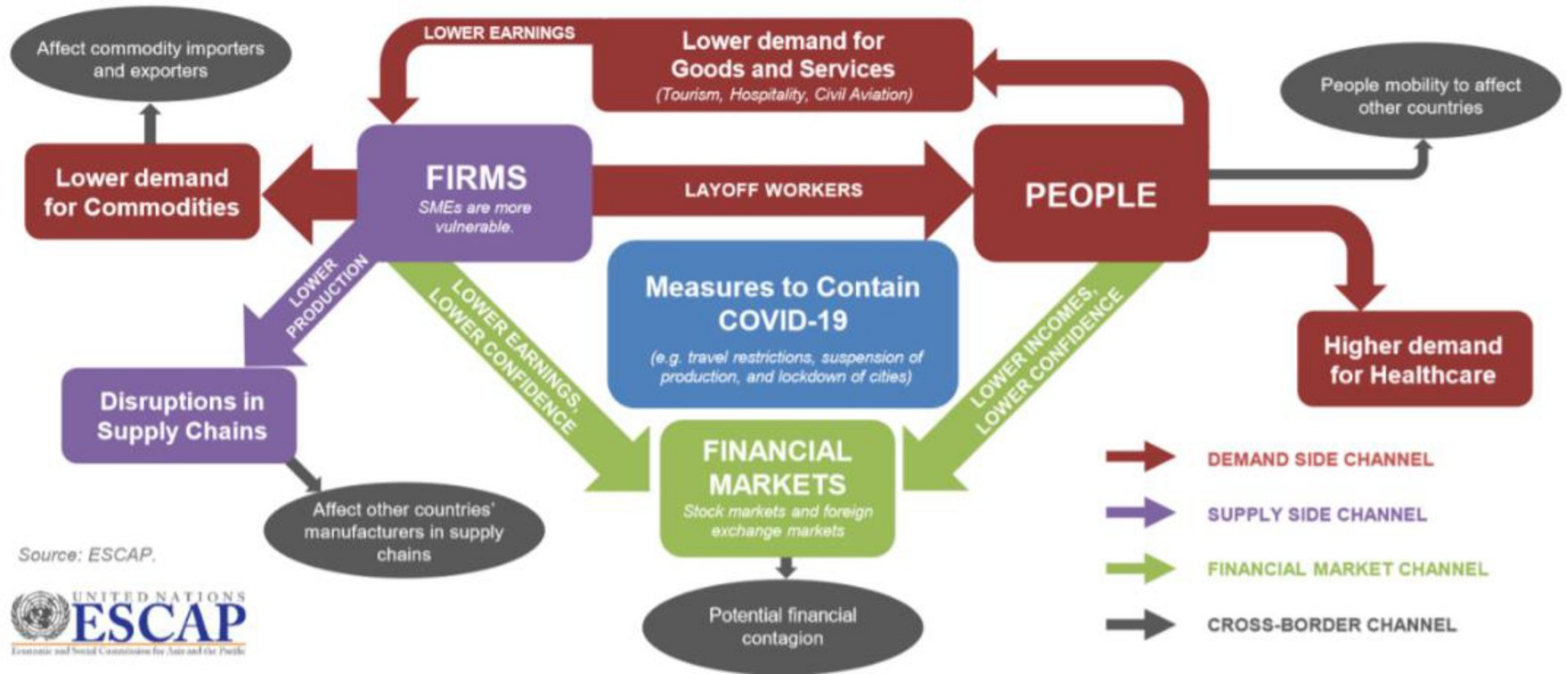
ESCAP sustainable business network response: COVID-19 and MSMEs. Source: Mikic (2020) [40].

Lockdowns and quarantines used to contain the disease resulted in further and more severe drops in capacity utilization [22]. Furthermore, supply chains are disrupted, resulting in shortages parts and intermediate goods. As illustrated in Fig 1, the pandemic had an impact on both the supply and demand sides of the global economy.

Some businesses in Thailand have been hit harder than others. Especially service businesses with a large number of customers today, such as tourism hotels and accommodations, and restaurants, were affected before other businesses were affected since the start of 2020. Although the pattern and severity of these effects can be predicted, it is difficult to predict how long they will last. Challenges posed by the COVID-19 pandemic that affects small and medium-sized businesses [23].

### 2.2 Business performance impact from demand side

On the demand side, a dramatic and unexpected loss of demand and revenue for MSMEs severely impairs their ability to function and/or causes severe liquidity shortages. Furthermore, consumers face income loss, fear of contagion, and increased uncertainty, all of which reduce spending and consumption. These effects are exacerbated because workers are laid off and the company is unable to pay wages. Some industries, such as tourism management and transportation, are particularly hard hit, contributing to a dropin business and consumer confidence. MSMEs, in general, are more vulnerable to “social distancing” than other companies [7].

According to Juergensen, Guimón, and Narula (2020) MSMEs’ demand has declined significantly as a result of lockdown measures, a drop in consumer confidence, and the closure of a number of GVCs in affected industries [24]. The magnitude of these demand and supply shocks is likely to differ depending on whether the firm is a stand-alone, specialized supplier, or knowledge-based MSMEs because of the liquidity squeeze and high volatility and pressure in all firms, particularly highly leveraged corporates, financial channels are under stress, affecting credit to MSMEs. The general panic caused by extreme circumstances, such as uncertainty about the length of the outbreak and the extent of its impact on human health and the economy, is exacerbating these trends.

### 2.3 Business performance impacts from management & operational side

From a management standpoint, several MSMEs temporarily closed down as a consequence of COVID-19 and the subsequent lockdown. For example, 46% of businesses said the COVID-19 outbreak has impacted their typical sales or revenue by 100%, and 79% of MSMEs’ owners have abandoned their business plans as a result. On the other hand, digitalisation opens up new prospects, and the COVID-19 epidemic will hasten MSMEs’ ability to take advantage of them. Unfortunately, due to ongoing financial concerns following the recession and the need to teach employees, some MSMEs may find it difficult to execute digital transactions. However, as well as digitisation, the consequences of the COVID-19 pandemic may provide a boost for other developments impacting manufacturing MSMEs, including the move to sustainable development.

### 2.4 Business performance impacts from policy initiatives

To improve the performance of business, the Thai government and other policy institutions took several initiatives based on the financial and non-financial benefits of MEMEs. Some major policy initiatives are discussed below.

#### 2.4.1 Amazing Thailand Safety and Health Administration (SHA)

To prepare for the return of tourism following COVID-19, Thailand’s Tourism Authority (TAT) has launched a new safety initiative. TAT launched the Amazing Thailand Safety and Health Administration (SHA) initiative in collaboration with public and private sector partners [25]. The SHA scheme is part of Thailand’s ongoing efforts to raise safety standards in the travel and tourism industry and regain the trust of domestic and international tourists in the aftermath of the worldwide COVID-19 crisis. Hotels, restaurants, tourist attractions, transportation services, and other facilities in Thailand may apply for SHA certification but must strictly adhere to the Ministry of Public Health’s COVID-19 control guidelines.

#### 2.4.2 DMHTT precautions

According to the WHO Thailand Situation Report, Thailand’s Centre for COVID-19 Situation Administration (CCSA) has advised the public to take DMHTT precautions to prevent COVID-19 spread: D–Distancing, M–Mask wearing, H–Handwashing, T–Temperature check, and T–Thai Chana contact tracing application. To cover all contingencies, the TAT would like to add E–Exercise, N–News, and E–Emergency [26]. However, as the COVID-19 situation worsened, the policy was implemented in all provinces of Thailand.

#### 2.4.3 Land and Building Tax

The goal of the Land and Building Tax Reduction Policy is to mitigate the economic and social impacts on land and building taxpayers. Because of the inability to engage in normal economic activities Because of the COVID-19 epidemic, the tax is reduced by 90% of the tax amount calculated for the 2021 tax year (Ministry of Finance, 2021).

#### 2.4.4 Tax date extension policies

Extension of deadlines for filing returns and payment of personal income tax, income tax, withholding, and VAT (extension of the form submission period) aims to alleviate tax burdens for income earners and entrepreneurs in the context of the COVID-19 epidemic, which will the liquidity of income earners and entrepreneurs by summarizing the details of the policies [27].

#### 2.4.5 Soft loan

To assist MSMEs in navigating this difficult period, the Bank of Thailand has changed its soft loan criteria to be more lenient. So, for Soft Loan measures in this COVID-19 situation, the Bank of Thailand has opened offers for MSMEs, allowing both existing and new customers to apply for loans more easily. Soft loans have a repayment period of up to ten years and are not subject to a one-time fee. The interest rate for the first two years of the contract is a fixed rate of 2% per year, with interest exemption for the first six months with the Ministry of Finance’s support, and the first five years of the contract average no more than 5% per year [28].

#### 2.4.6 Suspend property, debt relief policies

These policies aim to assist entrepreneurs who have potential but require time to recover from the effects of the COVID-19 outbreak and want to reduce the financial burden caused by borrowing from existing financial institutions and be able to resume regular business operations in the future. The Bank of Thailand offers loans to financial institutions to facilitate the transfer of assets as collateral for the payment of outstanding debts owed to financial institutions. The policy allows business operators to transfer assets to pay debts to financial institutions with the condition that they repurchase the transferred price and have the right to lease the assets back in order to prevent a lack of liquidity or debt default, which will affect the financial status of MSMEs and the stability of the country’s economy [29].

#### 2.4.7 Other financial relief packages

- *We Will Not Leave Each Other’ Programs*: The financial program is similar to another financial campaign launched by the Thai government earlier in 2020, which provides 15,000 bath in financial aid to individuals.
- *50 50 Co-Payment Programs*: According to The Bangkok Post (2020), the 50:50 campaign was introduced as a measure to help the general public reduce living expenses and vendors generate more revenue. It provides a subsidy on half the purchase price at participating vendors, up to 150 baht per day and up to 3,000 baht per person throughout the campaign.
- *We Win’ Programs*: The Thai government provided each of the 33.5 million recipients with 7,000 baht wired into their “Pao Tang” accounts in seven weekly instalments of 1,000 baht under the scheme. The extension of the deadline until the end of June will also help to lessen the impact of the new COVID-19 outbreak (Ministry of Finance, 2021).

## 3.0 Methodology

Initially, this study conducted a literature review to develop the variables, questionnaires, and analytical framework. The questionnaire was designed to collect data for empirically testable parameters and was structured based on the objectives. The items in the questionnaire were devised by the researchers and then validated by four experts. After finalising the questionnaire, it was approved by the ethical committee at Prince of Songkla University. Upon approval from the Ethical Committee, the address of the sample was contacted for help on … the online data collection. Email letters were written to these agencies to inform them of the research and how this study will need them to use their network coverage to assist us in the data collection process. Upon receiving permission from the agencies, the links to the online survey were shared with them. The data collection process covered the period between September 2022 and October 2022. This study distributed surveys among the owners and employees of MSMEs from the service sector, manufacturing sector, and retail sector in Thailand. A total of 403 MSMEs attended in the online questionnaire survey.

To address the research objectives, this study utilized the path modeling also know as, structural equation modeling (SEM). Due to the exploratory nature of this research, partial least square structural equation modeling (PLS-SEM) was used mostly to analyze data obtained through questionnaires. According to Hair et al. (2017), PLS-SEM is a second-generation analysis technique that is primarily for exploratory studies and overcomes the weakness of first-generation techniques like regression because it does not use the linear summation of indicators to represent the variables but rather uses the weighted aggregate of indicators to represent variables [30].

Before the data is analyzed, data screening, handling for missing values, reliability analysis and data properties tests were performed. Missing values were checked whether they are systematic or not. Data were tested for normality, and outliers were checked to ensure they did not distort statistical results. A non-response bias test was conducted to avoid seriously affecting the statistical results. Cronbach’s alpha test was used to test the reliability of the questionnaire. Exploratory factor analysis and the average variance extracted (AVE) were also used to test the reliability of the constructs. Discriminate validity was also done using the Fornell-Larcker criterion.

## 4.0 Result and analysis

### 4.1 Descriptive characteristics of the business

The descriptive characteristics of the business are presented in Table 1. The majority of businesses that participated in this study were categorized as small businesses, and about 70% of all businesses involved were solely owned. Most of the businesses operated mortar only form of business and most had only one branch. In terms of education, most of the owners of the businesses interviewed had a degree.

**Table 1:** Descriptive characteristics of the business.

Classification	Description	Frequency	Percentage
Size of business entity	Micro	127	31.5%
	Small	235	58.3%
	Medium	41	10.2%
	Total	403	100.0%
Type of business entity	Family business	76	18.9%
	Partnership	23	5.7%
	Private limited company	20	5.0%
	Sole Proprietorship	284	70.5%
	Total	403	100.0%
Form of business	Click and Mortar	135	33.5%
	Click only	23	5.7%
	Mortar only	245	60.8%
	Total	403	100.0%
Number of branches	Only one	375	93.1%
	2–5 branches	22	5.5%
	More than 5	6	1.5%
	Total	403	100.0%
Entrepreneur living with family	No	100	24.8%
	Yes	303	75.2%
	Total	403	100.0%
Entrepreneur level of education	No formal education	9	2.2%
	Primary	12	3.0%
	Secondary	36	8.9%
	Diploma/certificate/training	86	21.3%
	Degree	252	62.5%
	Master or higher	8	2.0%
	Total	403	100.0%
Entrepreneur marital status	Divorced	14	3.5%
	Married	199	49.4%
	Single	190	47.1%
	Total	403	100.0%

### 4.2 Model reliability test

The results of the reliability and validity tests are presented in Tables 2 and 3 below. All constructs had Cronbach’s alpha reliability of 0.811 or above. The minimum AVE for any construct is 0.533. The discriminant validity test satisfied Fornell-Lercker’s criterion, indicating that each construct is distinct. The factor loading of items relating to each construct was all above the 0.3 required.

**Table 2:** Factor analysis and reliability test results.

Construct and items	Factor Loading	α	AVE
**Demand Shock**		0.874	0.663
We lost contact with our many customers due to COVID-19	0.847		
Our customer can’t come to our premises during lockdown due to COVID-19	0.793		
Even if we tried to follow rules of the government and partially open, still some customers aren’t unable to come to our business during COVID-19.	0.834		
My business is not able to acquire new customer after CVOID-19 in compare to before COVID-19 normal time	0.854		
Customer’s overall purchasing ability decreased after CVOID-19 in compare to before COVID-19 normal time	0.738		
**Supply Shock**		0.878	0.601
Our ability to purchase inputs and materials for the business has decreased after CVOID-19 in compare to before COVID-19 normal time	0.812		
Overall supply prices increased after CVOID-19 in compare to before COVID-19 normal time	0.836		
The business is facing logistic problems after CVOID-19 in compare to before COVID-19 normal time	0.801		
Supplier take more time to deliver after CVOID-19 in compare to before COVID-19 normal time	0.775		
Supplies’ service quality has decreased after CVOID-19 in compare to before COVID-19 normal time	0.747		
Number of suppliers of my business has decreased after CVOID-19 in compared to before COVID-19 normal time	0.669		
**Management Shock**		0.823	0.586
Many structural changes in the business were needed due to adjustment of COVID-19	0.797		
The business procedure becomes complex due to the hygiene maintenance activities for COVID-19.	0.683		
Employee availability has decreased after CVOID-19 in compared to before COVID-19 normal time	0.774		
Employee effort/ output has decreased after CVOID-19 in compared to before COVID-19 normal time	0.792		
Many financial and accounting procedures were needed to change due to COVID-19 case	0.775		
**Policy Initiative**		0.893	0.701
The financial assistance programs of government is adequate to overcome COVID-19 effects on businesses.	0.858		
The financial assistance programs of government is adequate for business expansion and employment creation for MSMEs amid COVID-19 and post-covid-19 situation	0.793		
The financial assistance programs of government is adequate for financial leverage at post-COVID-19 situation	0.861		
The non-financial assistance, suggestion and supports you have received from government is adequate to survive in the COVID-19 and post-COVID-19 situation	0.838		
The financial assistance you have received from other agencies is adequate to survive in the COVID-19 situation	0.834		
**Business Vulnerability**		0.811	0.533
COVID-19 causes high level of disruption at my business work-flow and work schedule	0.732		
COVID-19 causes a possibility of my business shut down or failure	0.596		
COVID-19 causes instability of my business revenue and profit	0.811		
The potentiality of my business expansion is uncertain due to COVID-19	0.848		
The current supply of my business product or raw materials are more uncertain than before COVID-19	0.742		
My current business product stock out probability is more than before COVID-19	0.352		
The current customers of my business are more uncertain than before COVID-19	0.639		
The current customers’ demand changed such as deliver home or through post due to COVID-19	0.337		
**Business Adaptability**		0.849	0.589
My business can adapt with the coming recession due to COVID-19.	0.701		
My business can adapt with the changes of customer demand	0.703		
My business can survive with the low number of customers	0.626		
My business can bear the cost of delivery service of product	0.623		
My business can survive with low profit margin	0.538		
My business employees are able to work at any changes in business	0.832		
My business employees are committed to serve business in any circumstance	0.806		
My business has enough financial supports to survive in COVID-19 crisis time	0.719		
**Financial Performance**		0.849	0.567
The revenue of my business has relatively decreased after CVOID-19 in compared to before COVID-19 normal time	0.786		
Overall management costs of my business have relatively increased after CVOID-19 in compared to before COVID-19 normal time	0.743		
Transportation & delivery cost has relatively decreased after CVOID-19 in compared to before COVID-19 normal time	0.678		
The profitability of my business has relatively decreased after CVOID-19 in compared to before COVID-19 normal time	0.828		
The **s**ize of my business has relatively decreased after CVOID-19 in compared to before COVID-19 normal time	0.750		
My business is able to meet its financial obligations after COVID-19 compared to before COVID-19 normal time	0.725		
**Non-Financial Performance**		0.834	0.552
The service quality of my business relatively decreased after CVOID-19 in compared to before COVID-19 normal time	0.742		
Customer satisfaction of my business relatively decreased after CVOID-19 in compared to before COVID-19 normal time	0.844		
Customer loyalty of my business decreased after CVOID-19 in compared to before COVID-19 normal time	0.749		
The innovation and value creation of my business has decreased after CVOID-19 in compared to before COVID-19 normal time	0.592		
Competency and competitiveness of my business relatively decreased after CVOID-19 in compared to before COVID-19 normal time	0.815		
Inventory utilization of my business relatively decreased after CVOID-19 in compared to before COVID-19 normal time	0.687		

**Table 3:** Discriminant validity test (Fornell-Larcker Criterion).

Construct	1	2	3	4	5	6	7	8
Adaptability	0.700							
Demand Shock	-0.337	0.814						
Financial Performance	0.519	-0.370	0.753					
Management Shock	-0.125	0.555	-0.071	0.765				
Non-financial Performance	0.517	-0.284	0.447	0.008	0.743			
Policy Initiative	0.281	-0.204	0.371	-0.074	0.111	0.837		
Supply Shock	-0.344	0.337	-0.232	0.247	-0.097	-0.178	0.775	
Vulnerability	-0.277	0.653	-0.324	0.501	-0.112	-0.181	0.312	0.658

### 4.3 Structural model: Hypotheses testing

**Fig 2:**
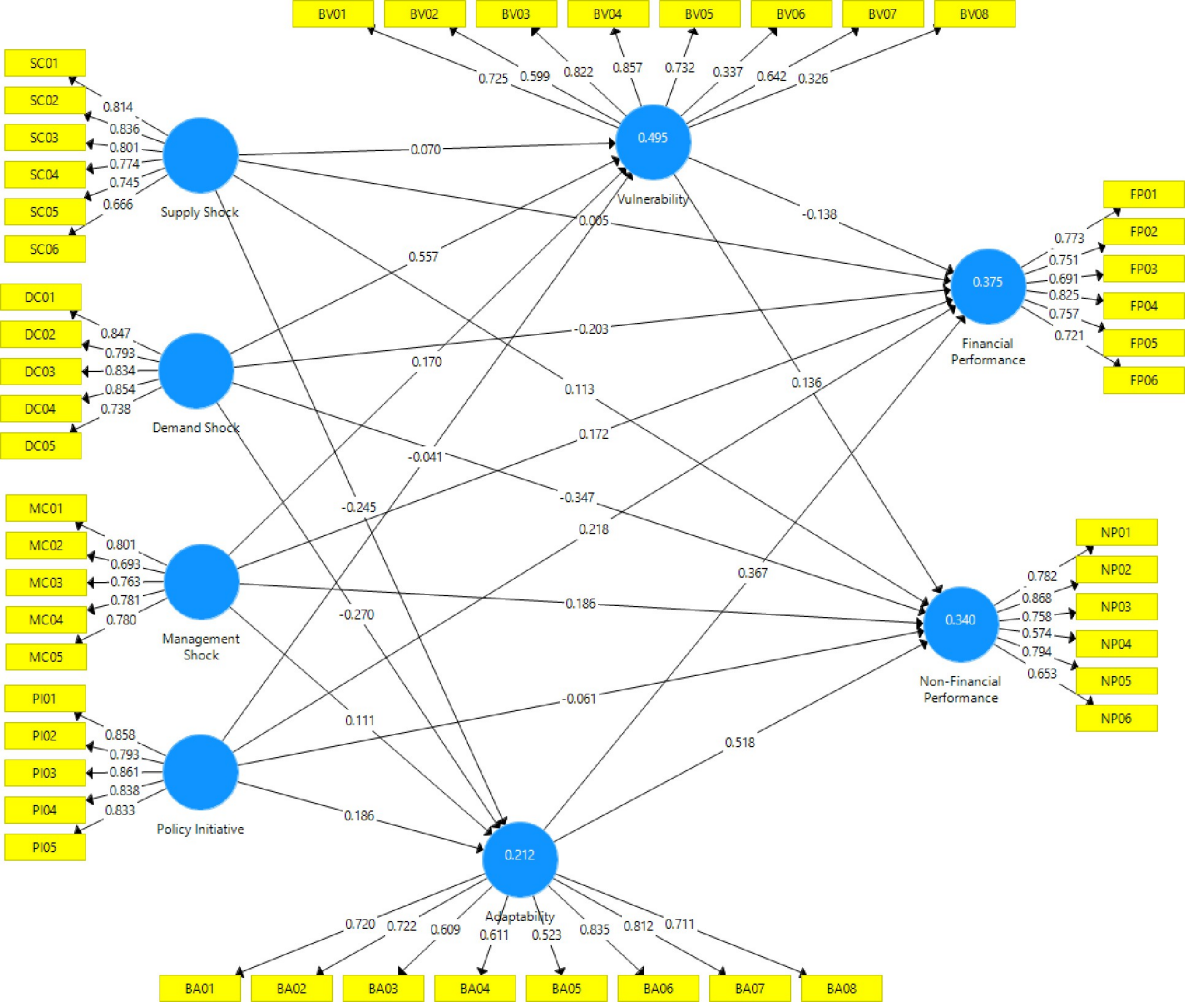
Result of structural model of constructs.

The structural relationship among construct and their path coefficient are represented in Fig 2 and summarized in Table 4 below.

**Table 4:** Results on impact of COVID 19.

Paths	Path Coefficients	Standard Dev	T-statistics
Adaptability -> Financial Performance	0.367**	0.060	6.140
Adaptability -> Non-Financial Performance	0.518**	0.057	9.023
Demand Shock -> Adaptability	-0.270**	0.067	4.027
Demand Shock -> Financial Performance	-0.203**	0.068	2.993
Demand Shock -> Non-Financial Performance	-0.347**	0.075	4.624
Demand Shock -> Vulnerability	0.557**	0.043	12.922
Management Shock -> Adaptability	0.111	0.081	1.367
Management Shock -> Financial Performance	0.172**	0.064	2.715
Management Shock -> Non-Financial Performance	0.186*	0.081	2.303
Management Shock -> Vulnerability	0.170**	0.050	3.402
Policy Initiative -> Adaptability	0.186**	0.069	2.704
Policy Initiative -> Financial Performance	0.218**	0.061	3.542
Policy Initiative -> Non-Financial Performance	-0.061	0.055	1.108
Policy Initiative -> Vulnerability	-0.041	0.045	0.908
Supply Shock -> Adaptability	-0.245**	0.063	3.917
Supply Shock -> Financial Performance	0.005	0.057	0.097
Supply Shock -> Non-Financial Performance	0.113	0.059	1.926
Supply Shock -> Vulnerability	0.070	0.049	1.429
Vulnerability -> Financial Performance	-0.138*	0.067	2.068
Vulnerability -> Non-Financial Performance	0.136	0.075	1.811

** Significant at *P< 0*.*01*

* Significant at *P <0*.*05*

Based on the findings in Table 4, MSMEs’ adaptability to COVID-19 has a significant effect both on short-term financial and non-financial performance. Adaptability, however, affect non-financial performance more than financial performance. The impact of demand shocks from lockdowns and other COVID-19 preventive measures is felt negatively and significantly by MSMEs, affecting their adaptability, financial performance, and non-financial performance. Demand shocks also significantly increased the vulnerability of MSMEs. Management shock positively affects MSMEs’ financial and non-financial performance and also their vulnerability. In terms of government policy initiatives, they tend to enhance only the adaptability and financial performance of MSMEs. The impact of policy initiatives is not significantly felt by the non-financial performance or vulnerability of MSMEs. This might be because most of the initiatives come in the form of financial assistance to businesses or their customers. From the supplier side, the supply shock brought on by COVID 19 only negatively impacted MSMEs’ ability to adapt. Finally, when MSMEs become vulnerable due to the impacts of COVID-19, it negatively affects their financial performance.

## 5.0 Discussions

The COVID-19 pandemic has already had a significant impact on MSMEs and will continue to have an impact for at least the next few months. Many individuals will be out of work if this industry collapses, which will have a significant impact on crime rates, as has been witnessed in many other counties around the country. Thailand is obviously not immune to the events that are taking place throughout the globe. Therefore, assisting MSMEs will help Thailand avoid social instability.

One of the ways MSMEs in Thailand can adapt to the changes brought by the pandemic is through technology solutions. MSMEs and their business models (reframed as depending on digitalization) are being adapted in order to ensure consistency and enhance business activity amid curfews. Even if the adoption of digital technologies to assist a company’s operations was not anticipated and was made on the spur of the moment, it results in increased competition and adaptability [31]. According to Kreutzer (2017), digital Darwinism condemns firms to a halt if they do not adjust to changes quicker than accessible technologies and the environment [32]. This indicates that MSMEs must take full advantage of their benefits in terms of capacity and agility in order to embrace innovative techniques and business models for long-term success [33]. They will be able to survive as a result of this.

Every disaster, irrespective of its cause, has a direct impact on financial markets. As a result, obtaining the necessary money may be one of the most difficult challenges facing MSMEs in Thailand when they need to modify their old business model and contemplate a different one. Companies could struggle financially if the pandemic continues [34]. Access to external financing is a problem that MSMEs encounter in a very special and unique way. This has to do with their lack of transparency and company model. A lack of tangible and physical resources that can be used as security against debts exacerbates these issues. This implies that many MSMEs are unduly dependent on self-generated cash to fund their day-to-day operations and leverage their activities.

Because of the COVID-19 pandemic’s unpredictability, new perspectives on the entrepreneurship paradigm have emerged [35, 36]. This is particularly true when the epidemic is combined with fast-paced digital change [37]. This is vital to assess because MSMEs are critical to supporting innovation, economic growth, and job creation [38].

Operational industry competition forces MSMEs to constantly develop fresh, innovative value offerings and become more robust in comparison to industry monopolies [31]. As a result, businesses must evaluate the possibility of collaborating with other MSMEs in the same discipline or area of work [39]. They will be able to do so by utilizing digital technologies. During the COVID-19 pandemic, MSMEs in any industry, particularly in Thailand, can be prompted to develop new strategies and make room for long-term performance and market dominance [31]. Implementing digital technology may also assist MSMEs in digitizing internal activities and operations, improving operational productivity, reengineering business models, ensuring corporate survival, and even enhancing business process innovation [31].

Finally, the study recommends that Thai MSMEs find the right balance between maintaining people’s health and economic well-being, which is crucial for policymakers as control practices are adopted. Even though there appears to be no one-size-fits-all strategy for dealing with the outbreak in all nations, Thailand and other nations must continue to monitor the scenario. Nonetheless, there are a number of repercussions that must be addressed in the next stage. This dilemma also serves as a cautionary tale for discussions leading up to the creation and acceptance of standards for an ethical working environment. The national plan for safety, healthcare, technological innovation, and utilization must be examined and updated as a top priority to align with the international new normal.

## 6.0 Conclusion

The purpose of this study is to assess the impact of COVID-19 and government initiatives on the financial and non-financial business performances of MSMEs’ in Thailand. According to the findings, MSMEs’ ability to respond to COVID-19 consequences has a significant impact on both financial and non-financial performance.

The results also showed that the non-financial performances experienced a greater impact in terms of adaptability compared to the financial performances. The impact of demand shock from lockdowns and other COVID-19 safety measures affected MSMEs’ adaptability for both financial performance and non-financial performance in a significantly negative way which also results to MSMEs vulnerability spiking up because of the demand shock. On the other hand, this study has showed that proper management of the demand shock has positively affected MSMEs’ financial and non-financial performances, as well as their vulnerability. In regards to the government policy measures, they tend to focus on improving MSMEs’ agility and financial performance but these legislative initiatives does not provide much solutions to the non-financial performances or vulnerability of MSMEs. This could be due to the fact that the majority of the projects is focused in providing financial aid to businesses or their customers. From the standpoint of suppliers, COVID-19’s supply shock only affected MSMEs’ ability to adjust. Lastly, when MSMEs become venerable and unstable as a result of COVID-19’s effects, it has a detrimental influence on their financial performance.

While this study has major practical and theoretical implications for how COVID-19 has affected MSMEs in Thailand, there are numerous flaws that should be considered before applying the results as solutions to the problems discussed. The sample size of the study may not be sufficient to cover all business concerns or all types of businesses. This means that it would advisable for future studies to use papers with larger sample sizes and that encompass all economic sectors. Aside from having disastrous economic consequences, COVID-19 has introduced additional challenges in terms of customer and manager/employee safety and health, influenced the creation of working systems and environments that now functions in a variety of ways. Lastly, future studies could look into these issues in order to better understand how the coronavirus pandemic has affected Thai businesses.

## Supporting information

S1 Data (XLSX)

## References

[1] Zhu N., Zhang D., Wang W., Li X., Yang B., Song J., et al. (2020). A novel coronavirus from patients with pneumonia in China, 2019. *New England Journal of Medicine*. doi: 10.1056/NEJMoa2001017 31978945 PMC7092803

[2] Zhou P., Yang X. L., Wang X. G., Hu B., Zhang L., Zhang W., et al. (2020). Discovery of a novel coronavirus associated with the recent pneumonia outbreak in humans and its potential bat origin. BioRxiv. *DOI*, 10(2020.01), 22.

[3] Perlman S. (2020). Another decade, another coronavirus. N Engl J Med.382:760–762. doi: 10.1056/NEJMe2001126 31978944 PMC7121143

[4] Munster V. J., Koopmans M., van Doremalen N., van Riel D., & de Wit E. (2020). A novel coronavirus emerging in China—key questions for impact assessment. *New England Journal of Medicine*, 382(8), 692–694. doi: 10.1056/NEJMp2000929 31978293

[5] Wang C., Horby P. W., Hayden F. G., & Gao G. F. (2020). A novel coronavirus outbreak of global health concern. *The Lancet*, 395(10223), 470–473. doi: 10.1016/S0140-6736(20)30185-9 31986257 PMC7135038

[6] Brun-Buisson C. (2003). SARS: the challenge of emerging pathogens to the intensivist. Intensive Care Med 29:861–862. Available at: <https://www.reporteroindustrial.com/temas/Coronavirus,-impact-on-the-worlds-factory+133999> [Accessed April 2020]. doi: 10.1007/s00134-003-1823-y 12858876 PMC7080195

[7] OECD. (2020). New OECD outlook on the global economy. Retrieved from https://www.oecd.org/coronavirus

[8] Sneader K., & Singhal S. (2020). Beyond coronavirus: The path to the next normal, Article McKinsey & Company. Retrieved from https://www.mckinsey.com/industries.

[9] Ernst and Young. (2020). Global Capital Confidence Barometer, 22nd ed. Retrieved from https://www.ey.com/en_my.

[10] Smith-Bingham R, & Hariharan K. (2020). This is the impact of the Coronavirus on business. World Economic Forum. Retrieved from https://www.weforum.org/agenda.

[11] Segal S., & Gerstel D. (2020). The Global Economic Impacts of COVID-19, Critical Questions, Center for Strategic and International Studies (CSIS). Retrieved from https://www.csis.org/analysis

[12] Humphries J. E., Neilson C., & Ulyssea G. (2020). The evolving impacts of COVID-19 on small businesses since the CARES Act.

[13] ITC (2020). MSME Competitiveness Outlook 2020: COVID-19: The Great Lockdown and its Impact on Small Business, ITC, Geneva, http://www.intracen.org (accessed on 23 June 2020).

[14] Buffington C., Dennis C., Dinlersoz E., Foster L., & Klimek S. (2020). Measuring the Effect of COVID-19 on US Small Businesses: The Small Business Pulse Survey (No. 20–16).

[15] Craven M., Liu L., Mysore M., & Wilson M. (2020). COVID-19: Implications for business. Executive Briefing, COVID-19: Briefing note, McKinsey & Company. Retrieved from https://www.mckinsey.com/business-functions/risk/our-insights

[16] Cassia L., & Minola T. (2012). Hyper-growth of MSMEs towards a reconciliation of entrepreneurial orientation and strategic resources. *International Journal of Entrepreneurial Behavior & Research*, 18(2), 179–197.

[17] Syed, H.A. (2019). Sustainability in Crisis: Towards Business Continuity in Small and Medium Enterprises. In: Proceedings of the 17th European Conference on Computer Supported Cooperative Work: The International Venue on Practice-centred Computing and the Design of Cooperation Technologies—Doctoral Colloquium Papers, Reports of the European Society for Socially Embedded Technologies, doi: 10.18420/ecscw2019_dc10

[18] Chittithaworn C., Islam M. A., Keawchana T., & Yusuf D. H. M. (2011). Factors affecting business success of small & medium enterprises (SMEs) in Thailand. *Asian Social Science*, 7(5), 180–190.

[19] Swierczek F. W., & Ha T. T. (2003). Entrepreneurial orientation, uncertainty avoidance and firm performance: an analysis of Thai and Vietnamese MSMEs. *The International Journal of Entrepreneurship and Innovation*, 4(1), 46–58.

[20] Office of Small and Medium Enterprises Promotion. (2011). Small and medium enterprises’ promotion plan (3rd) (2012–2016). Bangkok: Office of Small and Medium Enterprises Promotion.http://www.sme.go.th/upload/mod_download/report01- 20171024002621.pdf

[21] Papanikolaou D., & Schmidt D.W., L. (2020). The supply-side impact of COVID-19. Retrieved 7 August 2021, from https://voxeu.org/article/supply-side-impact-covid-19

[22] Cusmono L., & Rase S. (2020). Coronavirus (COVID-19): MSME policy responses. Retrieved 8 August 2021, from https://www.oecd.org/coronavirus/policy-responses/coronavirus-covid-19-sme-policy-responses-04440101/

[23] Bhundarak K. (2020). Covid-19 Business Survival by TBS. Retrieved 8 August 2021, from https://www.tbs.tu.ac.th/tbs-insights-2020/

[24] Juergensen J., Guimón J., & Narula R. (2020). European MSMEs amidst the COVID-19 crisis: assessing impact and policy responses. *Journal Of Industrial And Business Economics*, 47(3), 499–510. doi: 10.1007/s40812-020-00169-4

[25] Kongsuk P. (2020). Get to know SHA, the new normal travel safety badge. Retrieved 10 August 2021, from https://thestandard.co/sha-new-security-sign-for-new-normal-tourism/

[26] World Health Organization. (2021). WHO Coronavirus (COVID-19) Dashboard. [online] Available at: <https://covid19.who.int/> [Accessed 7 August 2021].

[27] KMPG, (2021). Retrieved 14 August 2021, from https://home.kpmg/us/en/home/insights/2021/05/tnf-thailand-extended-deadline-corporate-income-tax-return-filing-payment.html

[28] Bank of Thailand (2021). Retrieved 11 August 2021, from https://www.bot.or.th/covid19/content/sme/Pages/asset-warehousing.aspx

[29] BOT, (2021). Suspend Property, Debt Relief Policies. Retrieved on 11 August 2021, from https://www.bot.or.th/covid19/content/sme/Pages/asset-warehousing.aspx

[30] Hair J., Hollingsworth C. L., Randolph A. B., & Chong A. Y. L. (2017). An updated and expanded assessment of PLS-SEM in information systems research. *Industrial management & data systems*, 117(3), 442–458.

[31] Akpan I. J., Soopramanien D., & Kwak D. H. (2021). Cutting-edge technologies for small business and innovation in the era of COVID-19 global health pandemic. *Journal of Small Business & Entrepreneurship*, 33(6), 607–617.

[32] Kreutzer R. T. (2017). Treiber und Hintergründe der digitalen Transformation. In *Digitale transformation von geschäftsmodellen* (pp. 33–58). Springer Gabler, Wiesbaden.

[33] Papadopoulos T., Baltas K. N., & Balta M. E. (2020). The use of digital technologies by small and medium enterprises during COVID-19: Implications for theory and practice. *International Journal of Information Management*, 55, 10219236. doi: 10.1016/j.ijinfomgt.2020.102192 32836646 PMC7327446

[34] Cowling M., Brown R., & Rocha A. (2020). <? Covid19?> did you save some cash for a rainy COVID-19 day? The crisis and MSMEs. *International Small Business Journal*, 38(7), 593–604. 35125601 10.1177/0266242620945102 PMC8685737

[35] Ratten V. (2020). Coronavirus and international business: An entrepreneurial ecosystem perspective. *Thunderbird International Business Review*, 62(5), 629–634.

[36] Amankwah-Amoah J., Khan Z., & Wood G. (2021). COVID-19 and business failures: The paradoxes of experience, scale, and scope for theory and practice. *European Management Journal*, 39(2), 179–184. 10.1016/j.emj.2020.09.002 PMC7474582 38620607

[37] Zemtsov S. (2020). New technologies, potential unemployment and ‘nescience economy’during and after the 2020 economic crisis. *Regional Science Policy & Practice*, 12(4), 723–743. 10.1111/rsp3.12286 PMC7267282 38607788

[38] Dhewanto, W., Nazmuzzaman, E., & Fauzan, T. R. (2020, September). Cross-Countries’ Policies Comparison of Supporting Small and Medium-Sized Enterprises During Covid-19 Pandemic. In ECIE 2020 16th European Conference on Innovation and Entrepreneurship (p. 218). Academic Conferences limited.

[39] Hagemans I.; van Hemert P.; Meerkerk J.; Risselada A.; van Winden W. Interreg Europe—ABCitiEs—Policy Evaluation Report—AUAS. Amsterdam University of Applied Sciences, Municipality of Amsterdam, Mykolas Romeris University, Sunrise Valley Science and Technology Park, Manchester Metropolitan University, Manchester City Council, Faculty of Organization and Informatics of the University of Zagreb, City of Varaždin, City of Čakovec, Harokopio University and Athens Munici-Pality. 2020. Available online: https://www.interregeurope.eu/fileadmin/user_upload/tx_tevprojects/library/file_1601985586.pdf (accessed on 16 December 2020).

[40] Mikic M., & Anukoonwattaka W. (2020). Beyond the COVID-19 pandemic: coping with the ’new normal’ in supply chains.

